# Data Analysis and Classification of Autism Spectrum Disorder Using Principal Component Analysis

**DOI:** 10.1155/2020/3407907

**Published:** 2020-01-07

**Authors:** Ammar I. Shihab, Faten A. Dawood, Ali H. Kashmar

**Affiliations:** Department of Computer Science, College of Science, University of Baghdad, Baghdad, Iraq

## Abstract

Autism spectrum disorder (ASD) is an early developmental disorder characterized by mutation of enculturation associated with attention deficit disorder in the visual perception of emotional expressions. An estimated one in more than 100 people has autism. Autism affects almost four times as many boys than girls. Data analysis and classification of ASD is still challenging due to unsolved issues arising from many severity levels and range of signs and symptoms. To understanding the functions which involved in autism, neuroscience technology analyzed responses to stimuli of autistic audio and video. The study focuses on analyzing the data set of adults and children with ASD using practical component analysis method. To satisfy this aim, the proposed method consists of three main stages including: (1) data set preparation, (2) Data analysis, and (3) Unsupervised Classification. The experimental results were performed to classify adults and children with ASD. The classification results in adults give a sensitivity of 78.6% and specificity of 82.47%, while the classification results in children give a sensitivity of 87.5% and specificity of 95.7%.

## 1. Introduction

Autism spectrum disorder (ASD) is a condition that can be characterized by a constant deficit in social communication, social interaction, and the presence of restrictive and repetitive behavior. It is an early developmental disorder characterized by alterations in socialization associated with a deficit in the visual perception of faces and emotional expressions. This deficit in the perception of faces and emotional expressions seems to be linked to the peculiarities of the gaze in autistic pathology [1]. The study of this behavioral disorder is carried out by the measurement of different ocular parameters (fixation time, distance and speed of exploration, ocular path) during the perception of neutral (with direct or deviant gaze) and emotional faces (expressing joy or sadness) [2].

Some symptoms in ASD typically appear after 2 years of age [3]; therefore, the early diagnosis could be a better opportunity to get treatment and healing [4]. It is generally recognized that traditional clinical methods have difficulty in well distinguishing patients from healthy controls (HC) [5]. Therefore, data analysis and classification of ASD is still challenging due to unsolved issues arising from many severity levels and range of signs and symptoms. The commonly used tools for analyzing the dataset of autism are functional magnetic resonance imaging (fMRI), Electroencephalography (EEG), and more recently “eye tracking”. Eye tracking is a system of monitoring of the gaze grouping together a set of techniques which make it possible to record the ocular movements and to measure several parameters such as the time of fixation of the image, the number of fixations of an area of the image, etc. The objective of eye tracking system is to examine perceptual characteristics of ASD and facilitate study into the abnormal behavior of visual attention and oculomotor patterns that contribute to clinical characteristics of ASD. The detailed and objective measures of pupil eye behavior, eye tracking system used to identify disorder specific characteristics, enhance early identification, and inform treatment. Particularly, examiners of ASD have benefited from integrating eye tracking into their research paradigms.

**Figure 1:**
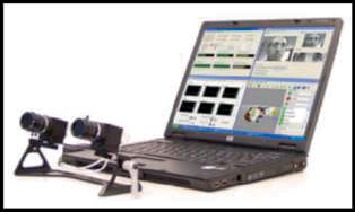
Monitoring system [7].

Eye tracking technique has been largely applied in these studies to reveal mechanisms underlying impaired task performance and abnormal brain functioning, essentially through the processing of social information. While older children and adults with ASD comprise the superiority of research in this area, eye tracking is useful for studying young children with the disorder as it offers an extensive tool for assessing and quantifying early emerging developmental abnormalities. Implementing eye tracking of children with ASD, therefore, is associated with a number of challenges, including problems with compliant behavior resulting from the given task requested and disorder related psychosocial considerations [6]. The eye tracking implementation includes: (1) Eye tracking equipment, (2) Testing environment and stimuli, (3) Procedures & analysis, and (4) Representative results. The recordings in step1 have been carried out using a look-up system comprising a computer equipped with two analogue cameras as illustrated in Figure 1. Following a projection of images representing neutral faces or deviated eyes, this system makes it possible to capture the directions, movements, and positions of the eyes during the projection and to superimpose them in order to calculate in real time the temporal and statistical measurements [7]:

- Fixing time of the image;
- The number of fixations of an area of the image;
- Nonimage fix time; the papillary diameter.

In order to produce interesting results, the eye tracking device can be a better implementation option for use in processes that possibly involve lack of perception, such as photographs or films which involve sense. The model involved in this test shows to a human face a screen (or a movie involving social interactions), and at the same time, catches the position and interest of the patient on the screen as data in order to analyze.

Classifying autism automatically according to time is interesting in more ways than one. It allows, in particular, to follow the evolution of the pathology following the medicated or nondrug therapy practice. One can, for example, judge its reeducation according to whether the position of the subject is close or not to the group of people without autism. The second point concerns the informative parameters that allowed this classification. The temporal follow-up and the connection of these parameters with neurophysiological information can certainly help in understanding the mechanisms put into action in people with autism [8].

This study is based on the classification of data provided by the follow-up material according to the two groups (autistic and control). The main aspect is to implement the Principal Component Analysis (PCA) which will allow us to reduce the size of the representation space and to retain only the parameters that provide discriminating information. Two ways will be followed, first concerns the development of classifiers based on statistical data already provided by the system “eye tracking”. Second finds a new descriptor using the eye trajectories. The second aspect of this study is directed towards searching for new parameters according to the analysis of trajectory. Given the complexity of the dynamics underlying the time series or trajectories, it is natural to turn to tools from the information theory or chaos theory. This assumption is realistic if we consider that the trajectory corresponds to the output of a nonlinear dynamic system (the brain) excited by an input—the visual stimulus. Therefore, the main contribution by using PCA is to decrease the dimensionality of a dataset consisting of a large number of consistent variables, while retaining the variation present in the data set by choosing a threshold to retain only those that express a significant difference.

## 2. Materials and Methods

**Figure 2:**
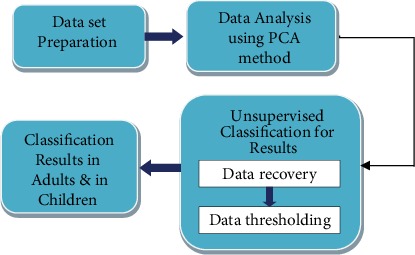
Block diagram of the proposed method.

This study aims to analyze and classify the dataset of ASD in adult and child patients. The framework of the proposed method is illustrated in a block diagram as shown in Figure 2. It consists of three main stages: (1) data set preparation, (2) Data analysis, and (3) Unsupervised Classification including data recovery and thresholding. In the first stage, dataset that is used in this study with their characteristics is explained. Establishing the mathematical foundations of Principal Component Analysis (PCA) which is considered as a method of reducing the size of data is presented in stage two. In the third stage, the unsupervised classification method is used to classify results in adults and children with ASD by using two steps including: data recovery and data thresholding.

### 2.1. Data Set Preparation

The dataset used in this work consists of two groups as presented in Table 1. The first group includes 30 adult patients with ASD (15 male, 15 female) and 36 adults without ASD (17 male, 19 female). Second group includes 14 child patients with ASD (9 male, 5 female) and 22 children without ASD (12 male, 10 female).

**Figure 3:**
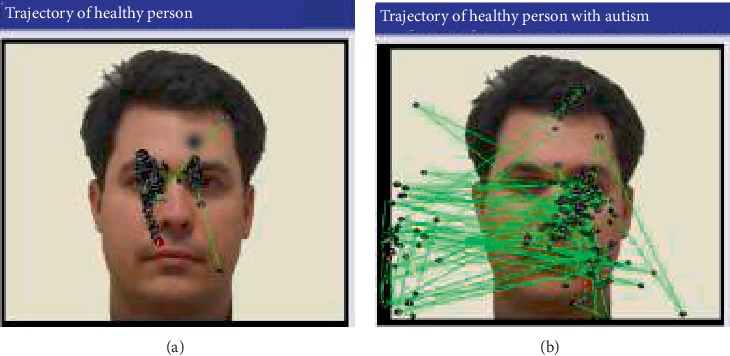
A preliminary study on eye tracking trajectories of (a) healthy person and (b) person with autism [7].

All datasets were used in the age range of 4 to 60 years. Each dataset includes five fields (*x*, *y*, distance, left diam, right diam, and time), where *x* and *y* were obtained from trajectory eye tracking system. The distance field represents the length of distance between the points (*x*, *y*) and the central point on screen (384, 512). Left diam and right diam represent the left eye and right eye, respectively. The time field represents the start and end of the experiment. A preliminary study on eye tracking trajectories of patients studied as seen in Figure 3 showed a rudimentary statistical analysis. Principal Component Analysis (PCA) provides interesting results on the statistical parameters that are studied such as the time spent in a region of interest, the attachment time. Some of the other studies, involving tools using Euclidean geometry and nonEuclidean, also show interesting results.

### 2.2. Data Analysis Using PCA Method

Principal Component Analysis (PCA) is a method of extracting important variables (in form of components) from a large set of variables available in a dataset. It elicits low from high dimensions of the featured dataset with a motive to possibly capture as much more information. In addition, with any variables, visualization becomes much meaningful. PCA is useful when dealing with three or higher dimensional data. It is carrying out symmetric correlation or covariance matrix. The inherent problem in multivariate statistics is one of the obstacles in visualizing data that have many variables. The datasets contain many variables, groups of variables are often moving together. More than one variable might be measuring the principle governing the system. The affluence of usefulness can enable to measure scores of variables. When it happens, the advantage can be taking it to redundancy of information, and the problem can be simplified using replacement of a group of variables with a single new variable.

PCA method can generate a new set of variables; it is called *principal components *[9]. Each principal component represents a linear combination of original variables. All principal components are perpendicular to each other, so there is no redundant information. The principal components as a whole form an orthogonal basis for the space of the data. The first principal component is a single axis in a matrix. And the variance of variable is the maximum of all possible choices of the first axis. The second principal component is related to another axis in a matrix, perpendicular to the first. The observation on this axis generates another new variable. The variance of this variable is the maximum of all possible choices in the second axis.

In this study, the main purpose of PCA is to decrease the dimensionality of a dataset consisting of a large number of consistent variables, while retaining the variation present in the dataset. Thus, from the matrix, *M* [*m* × *n*] of the data (*m* is the number of observations and *n* represents the number of parameters), we project the data in a reduced-size basis to establish two groups. To do this, we began by reducing the variables of the matrix *M*, by choosing a threshold to retain only those that express a significant difference [10, 11]. There are three roles of the PCA such as:

1. Study the linkage (correlation) between the variables;
2. Project the observations following new axes results of linear combinations of the initial variables, reduction of dimension and obtaining new coordinates;
3. Change to a new orthonormal basis to implement data variances.

## 3. Unsupervised Classification

Unsupervised classification is used when the class number is not known. There are two categories of unsupervised classifications: hierarchical and nonhierarchical. In the hierarchical classification (HC), the created subsets are nested hierarchically in one another. We distinguish the descending HC, which starts from the set of all the individuals and breaks them into a certain number of subsets, each subset then being divided into a certain number of subsets, and so on, and the ascending HC starts from the individuals that are grouped into subsets, which are in turn grouped, and so on. In nonhierarchical classification, individuals are not structured hierarchically. If each individual is only part of a subset, it is called partition. If each individual can belong to several groups, with the probability *P*(*i*) of belonging to group *i*, then we speak of overlap [12]. In this study, the unsupervised classification consists of two main steps such as illustrated in the following subsections.

### 3.1. Data Recovery

In order to implement this stage, data recovery contains 73 criteria in the file, 275 person record—some persons have 6 datasets and some others have 8 datasets. The number of patients participated in the experiment is 45. The file “Photo.txt” contained 275 records and the data file which is recorded is related with data of criteria used; Table 2 presents the criteria of time with sample datasets for three ASD patients.

Also, Table 3 presents the criteria of statistical measurements with sample dataset for three ASD patients.

Each element in the data record can be represented in one of criteria. For example, the first criterion in the file “crietria.txt” is Time span shown start (seconds) which represents the start time of experiment, and so on.

### 3.2. Data Thresholding

The main aim of data thresholding in the methodology is to transport dataset file based on the standard deviation matrix in order to reduce the size of the matrix by using threshold. Therefore, three main steps are implemented such as:

*First Step.*Suppose l = line and *c* = column in the dataset file. The standard deviation matrix on the datasets has been used in order to reduce the size of the matrix by removing the standard deviation data, and used threshold. For example, to find the low in autistic and healthy:
  For *i* = length(stdr):−1 : 1
   if ((stdr(*i*) < = 10))
    photo(*i*,:)=;
    criteres(*i*,:)=;
    stdr(*i*,:)=;
   end
  end
The line *I* in the matrix has been deleted, and the dataset matrix has been converting to transportation (*t*).
*Second Step.* It is used to center the matrix. The values in the datasets for the first line (*i*) have been done and extracting the mean, and medium value.  Table 4 shows (stdr, stdm, median, and variance) measurements.
*Third Step.* Is used to reduce the matrix by using standard division of the datasets values. Accordingly, the algorithm below can be given using Matlab:
*B* = repmat(*A*, *n*) returns an array containing *n* copies of *A* in the row and column dimensions. The size of *B* is size (*A*)^∗^*n* when *A* is a matrix.
*B* = repmat(*A*, *r*1,…, *rN*) specifies a list of scalars, *r*1,…, *rN*, that describes how copies of *A* are arranged in each dimension. When *A* has *N* dimensions, the size of *B* is size (*A*). ^∗^[*r*1 … *rN*]. For example, repmat([1 2; 3 4], 2, 3) returns a 4-by-6 matrix.
*Fourth Step.* Principal components analysis PCA has been used in order to describe the coefficient component values in a matrix:
  (1) $$\begin{array}{c} \text{COEFF}=\text{princomp}\left(X\right), \end{array}$$
performs principal components analysis (PCA) on the *n*-by-*p* data matrix *X*, and returns the principal component coefficients and observation of the importance of the main components [coefs, scores, variances] = princomp(photos).

*First Step.*Suppose l = line and *c* = column in the dataset file. The standard deviation matrix on the datasets has been used in order to reduce the size of the matrix by removing the standard deviation data, and used threshold. For example, to find the low in autistic and healthy:

  For *i* = length(stdr):−1 : 1
   if ((stdr(*i*) < = 10))
    photo(*i*,:)=;
    criteres(*i*,:)=;
    stdr(*i*,:)=;
   end
  end

## 4. The Experimental Results

### 4.1. Result of Classification in Adults

**Figure 4:**
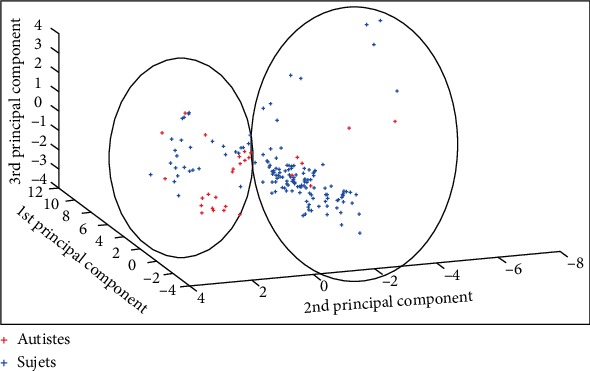
Classification results in adults.

For adult data, we have an *M* matrix [182, 73] (182 = 154 controls + 28 autistic). For a threshold, equal to 10, only 15 parameters are retained, the matrix becomes: *M* [182, 15]. The results of the manual classification are given in Figure 4. Two groups are formed:

- One group contains 6 autistic subjects out of 28 and 127 out of 154.
- A second group contains 22 autistics among 28 and 27 of 154.

In order to evaluate these results, two metrics, sensitivity and specificity, are used. We recall that sensitivity is defined as:

(2) $$\begin{array}{c} \text{Se}=\frac{\text{VP}}{\left(\text{VP}+\text{FN}\right)}, \end{array}$$

where VP indicates the true positive and FN the false negative. The specificity is defined by:

(3) $$\begin{array}{c} \text{Sp}=\frac{\text{VN}}{\left(\text{FP}+\text{VN}\right)}, \end{array}$$

where VN indicates the true Negative and FP false positives.

Thus, the test performed to classify patients with autism gives: a sensitivity of 78.6% and a specificity of 82.47%.

### 4.2. Result of Classification in Children

**Figure 5:**
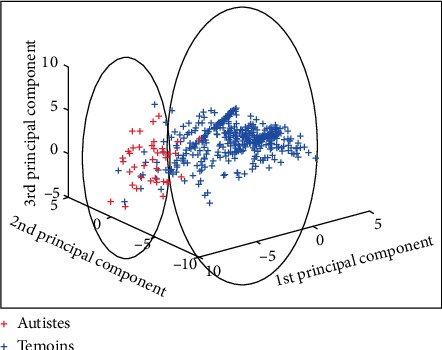
Classification in children.

For children, we have an *M* matrix [433, 73] (433 = 393 controls + 40 autistic). For a threshold equal to 10, only 19 parameters are retained, the matrix becomes: *M* [433, 19]. The results of the manual classification are shown in Figure 5. Two groups are formed:

- One group contains 5 autistics among 40 and 376 out of 393.
- A second group contains 35 autistic patients of 40 and 17 of 393.

Finally, the test performed to classify patients with autism gives: a sensitivity of 87.5% and a specificity of 95.7%.

Finally, thecomparison results of classification performance in both adults and children are presented in Table 5.

Comparison of methods in Table 5 shows that the proposed method obtained a sensitivity of 87.50% in children, the proposed method has a higher performance than the other methods with respect to children. As for specificity, the obtained result of 95.71% for children is considered approximately equal to the result of the SVM method. As a final result, the proposed method shows a higher percentage in children than in adults.

## 5. The Discussion for Results

**Figure 6:**
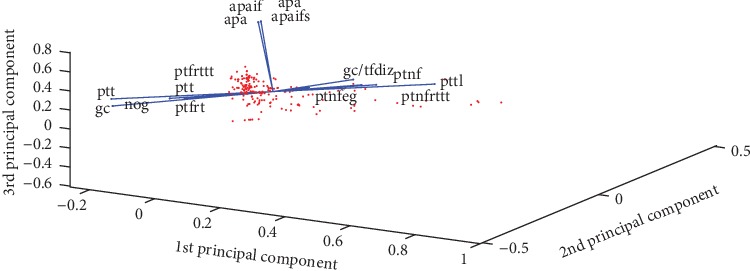
Adult correlation circle.

**Figure 7:**
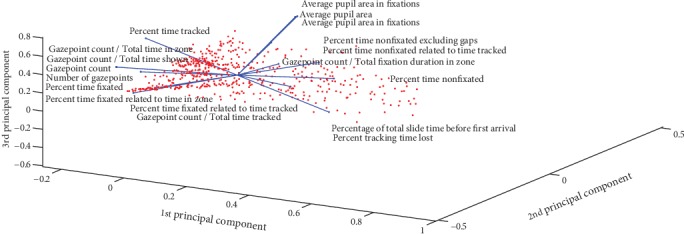
Children correlation circle.

The results obtained after applying the PCA method on the dataset record show a fairly good classification for adults and a very good classification for children. On the other hand, out of 73 criteria, only 15 were retained in adults and 19 in children. The correlation circle for adults and for children is shown in Figures 6 and 7, respectively. The following are some of the parameters selected by the most discriminating PCA method:

- Ptt: Percent time tracked.
- Pttl: Percent tracking time lost.
- Ptf: Percent time fixated.
- Ptneg: Percent time nonfixated excluding gaps.
- Ptfrttt: Percent time fixated related to time tracked.
- Ptnrttt: Percent time nonfixated related to time tracked.
- Apa: Average pupil area.
- Apaifs: Average pupil area in fixations.
- Nog: Number of gazepoints.
- Ptfrt: Percent time fixated related to time in zone.
- Ptnf: Percent time nonfixated.
- Ptfrtpfd: Percent time fixated related to total fixation duration.
- Apif: Average pupil area in fixations.
- Gc: Gazepoint count.
- Gc/ttiz: Gazepoint count/total time in zone.
- Gc/tfdiz: Gazepoint count/total fixation duration in zone.

As mentioned earlier, unsupervised classification is based on two main steps: (1) Data recovery that contains 73 criteria in the file, 275 person record—some persons have 6 datasets and some others have 8 datasets. The number of patients participated in the experiment is 45. Each element in the data record can be represented in one of criteria. (2) Data thresholding is applied for transportation dataset file based on the standard deviation matrix in order to reduce the size of the matrix by using threshold. For adult classification, a threshold equal to 10 and only 15 parameters are retained, while for children classification, a threshold equal to 10 and only 19 parameters are retained. Finally, for both children and adults, the performance of classifiers is good since there is on average 80% sensitivity and 90% specificity.

## 6. Conclusion

The main goal of this study is to analyze and classify the data set of autism specter disorder (ASD) in adult and child patients based on practical component analysis (PCA) method. The framework of this study consists of three main stages including: data set preparation, Data analysis and classification. Two groups of datasets were used in the age range of 4–60 years. The first group includes 30 adult patients with ASD (15 male, 15 female) and 36 adults without ASD (17 male, 19 female). Second group includes 14 child patients with ASD (9 male, 5 female) and 22 children without ASD (12 male, 10 female).

Unsupervised classification stage consists of three steps: Data recovery, Data thresholding, and Eentrance the PCA. Data recovery contains 73 criteria in the file, 275 person record—some persons have 6 datasets and some others have 8 datasets. The number of patients participated in the experiment is 45. Data thresholding is needed to transport dataset file based on the standard deviation matrix in order to reduce the size of the matrix by using threshold. For adult classification, a threshold equal to 10 and only 15 parameters are retained, while for children classification, a threshold equal to 10 and only 19 parameters are retained.

The main purpose of PCA is to decrease the dimensionality of a dataset consisting of a large number of consistent variables, while retaining the variation present in the data set. The results obtained after the applying PCA method on the dataset record show a fairly good classification for adults and a very good classification for children. On the other hand, out of 73 criteria, only 15 were retained in adults and 19 in children. To classify adult patients with autism, the test performed gives a sensitivity of 78.6% and a specificity of 82.47%, while the test performed to classify child patients with autism gives a sensitivity of 87.5% and a specificity 95.7%.

Finally, for both children and adults, the performance of classifiers is good since there is on average 80% sensitivity and 90% specificity. Future studies will use the neuron sign technologies to classify the signals obtained by the EEG device.

## Figures and Tables

**Table 1:** Data set in two groups (adults and children).

Data set	ASD	Without ASD
Adults (male : female)	30 (15 : 15)	36 (17 : 19)
Childern (male : female)	14 (9 : 5)	22 (12 : 10)

**Table 2:** Criteria (time) with sample datasets for three patients.

Criteria	Datasets patient 1	Datasets patient 2	Datasets patient 3
Time span shown start (seconds)	0	0	0
Time span shown end (seconds)	4.031	4.064	4.076
Total time shown (seconds)	4.031	4.064	4.076
Total time tracked (seconds)	4.031	4.064	4.076
Total tracking time lost (seconds)	0	0	0
Total fixation duration (seconds)	1.472	1.119	1.472
Total time nonfixated excluding gaps (seconds)	2.559	2.945	2.604
Percent time tracked	100	100	100
Percent tracking time lost	0	0	0
Percent time fixated	36.517	27.53445	36.11385
Percent time nonfixated excluding gaps	63.483	72.46555	63.88615
Percent time fixated related to time tracked	36.517	27.53445	36.11385
Percent time nonfixated related to time tracked	63.483	72.46555	63.88615

**Table 3:** Criteria (statistical measurements) with sample datasets for three patients.

Criteria	Datasets patient 1		Datasets patient 2		Datasets patient 3	
Average pupil *x* diameter		5.575926		5.719807		5.67561	
Average pupil *y* diameter		5.026389		3.678744		5.390244	
Average pupil area		22.13001		16.75918		24.32972	
Pupil *x* diameter std dev		0.522938		1.113485		0.63043	
Pupil *y* diameter std dev		0.828278		1.356835		0.632612	
Pupil area std dev		4.767189		7.08194		5.749157	
Number of fixations		21		13		18	
Fixation count/total time shown		5.209625		3.198819		4.416094	
Fixation count/total time tracked		5.209625		3.198819		4.416094	
Average fixation duration (seconds)		0.070095		0.086077		0.081778	
Stddev fixation duration (seconds)		0.020792		0.03601		0.029311	
Average pupil *x* diameter in fixations		5.620588		6.172973		5.753061	
Average pupil *y* diameter in fixations		4.929412		3.748649		5.477551	
Average pupil area in fixations		21.90268		18.16944		25.10493	
Pupil *x* diameter std dev in fixations		0.585525		0.480096		0.687923	
Pupil *y* diameter std dev in fixations		0.951454		1.085678		0.68316	
Pupil area std dev in fixations		5.42101		5.666592		6.214513	

**Table 4:** STDR, centering matrix of medium, STDM for reduction matrix, and variances.

STDR	Median	STDM	Variances
10.4977933688735	97.4305486607143	10.49779	9.95271791924796
10.4977933688735	2.56945133928571	10.49779	2.39932868236332
23.3795873638281	67.4581276453242	23.37959	1.85467953647792
19.9044233356702	29.9724210154945	19.90442	0.487037283967104
22.3894435735591	68.2123465458792	22.38944	0.289512288889336
22.3894435735604	31.7876534541209	22.38944	0.0133786232669430
11.1817998193019	22.7235711561703	11.1818	0.00334566578743095
11.2579070533580	22.7449549919121	11.25791	2.93277886896789e-21
36.3665144661355	184.961538461538	36.36651	1.03077453398389e-21
11.1817998193019	22.7235711561703	11.1818	1.09872800892096e-22
23.3795873638281	67.4581276453242	23.37959	2.48201346799659e-30
23.3795873641116	32.5418723547802	23.37959	8.13990675136350e-32
11.2579070533580	22.7449549919121	11.25791	1.03826922127465e-32
36.3665144661355	184.961538461538	36.36651	7.44794263958027e-34
85.0037111401057	89.7062333048352	85.00371	5.44491111294972e-34

**Table 5:** Comparison results of classification performance in both adults and children.

Classification method	Sensitivity		Specificity	
	Adults	Children	Adults	Children
SVM [7]	71.43%	70.00%	93.50%	95.91%
Decision tree [13]	80.07%	78.40%	81.11%	72.57%
Proposed method	78.60%	87.50%	82,47 %	95.71%

## Data Availability

The data used to support the findings of this study are available from the corresponding author upon request.
